# Prostate Cancer Detection from MRI Using Efficient Feature Extraction with Transfer Learning

**DOI:** 10.1155/2024/1588891

**Published:** 2024-05-16

**Authors:** Rafiqul Islam, Al Imran, Md. Fazle Rabbi

**Affiliations:** ^1^Department of IoT and Robotics Engineering, Bangabandhu Sheikh Mujibur Rahman Digital University, Gazipur, Bangladesh; ^2^Department of Computer Science and Engineering, Green University of Bangladesh, Dhaka, Bangladesh

## Abstract

Prostate cancer is a common cancer with significant implications for global health. Prompt and precise identification is crucial for efficient treatment strategizing and enhanced patient results. This research study investigates the utilization of machine learning techniques to diagnose prostate cancer. It emphasizes utilizing deep learning models, namely VGG16, VGG19, ResNet50, and ResNet50V2, to extract relevant features. The random forest approach then uses these features for classification. The study begins by doing a thorough comparison examination of the deep learning architectures outlined above to evaluate their effectiveness in extracting significant characteristics from prostate cancer imaging data. Key metrics such as sensitivity, specificity, and accuracy are used to assess the models' efficacy. With an accuracy of 99.64%, ResNet50 outperformed other tested models when it came to identifying important features in images of prostate cancer. Furthermore, the analysis of understanding factors aims to offer valuable insights into the decision-making process, thereby addressing a critical problem for clinical practice acceptance. The random forest classifier, a powerful ensemble learning method renowned for its adaptability and ability to handle intricate datasets, then uses the collected characteristics as input. The random forest model seeks to identify patterns in the feature space and produce precise predictions on the presence or absence of prostate cancer. In addition, the study tackles the restricted availability of datasets by utilizing transfer learning methods to refine the deep learning models using a small amount of annotated prostate cancer data. The objective of this method is to improve the ability of the models to generalize across different patient populations and clinical situations. This study's results are useful because they show how well VGG16, VGG19, ResNet50, and ResNet50V2 work for extracting features in the field of diagnosing prostate cancer, when used with random forest's classification abilities. The results of this work provide a basis for creating reliable and easily understandable machine learning-based diagnostic tools for detecting prostate cancer. This will enhance the possibility of an early and precise diagnosis in clinical settings such as index terms deep learning, machine learning, prostate cancer, cancer identification, and cancer classification.

## 1. Introduction

Innovative and precise diagnostic procedures are needed to improve patient outcomes for prostate cancer, the second most frequent malignancy in men worldwide. Prostate cancer identification must be nuanced because of its wide range of clinical manifestations and disease progression. While useful, traditional diagnostic procedures struggle to achieve the precision needed for prompt action. Using supervised machine learning algorithms to detect prostate cancer could improve diagnostic accuracy and clinical decision-making. Machine learning has revolutionized medical diagnoses. Supervised machine learning algorithms use advanced computational methods to find patterns and relationships in complicated datasets to diagnose prostate cancer. This study examines machine learning and prostate cancer diagnosis to create a reliable model that can distinguish between malignant and benign diseases [1]. Due to prostate cancer's heterogeneity, diagnosing and treating patients requires better diagnostic precision. Clinical records, imaging data, and pathology data can be used to train machine learning algorithms to find complex patterns in this heterogeneity. These algorithms systematically analyze and learn from many characteristics to improve prostate cancer diagnosis and help clinicians make informed decisions. The dataset and machine learning model are improved by adding modern imaging technologies such as magnetic resonance imaging (MRI). This comprehensive strategy, which includes several clinical and imaging characteristics, aims to improve prostate cancer identification by addressing diagnostic constraints. As science progresses, ethical issues surrounding medical machine learning must be examined. To seamlessly integrate these algorithms into established healthcare frameworks, interpretability, transparency, and appropriate deployment of advanced technology in clinical settings must be considered [2]. An explainable artificial intelligence (XAI) model was developed for diagnosing clinically significant prostate cancer (PCa) using biparametric MRI and Prostate Imaging Reporting and Data System (PI-RADS) features. XAI-assisted readings improved the confidence of nonexperts in assessing PI-RADS 3 lesions and reduced reading time by 58 seconds. [3]. By incorporating diagnostically relevant patient data such as PSA, previous biopsy status, and demographic information, the text-image fusion model achieves better risk assessment and localization of prostate cancer on MRI. The model outperforms traditional imaging-only baseline models in detecting prostate cancer and distinguishing aggressive from nonaggressive cancers. It achieves an AUC of 0.828 for identifying cancer in the prostate and 0.820 for detecting aggressive cancer, compared to baseline models [4]. The conventional method of transrectal ultrasound (TRUS-) guided biopsy for prostate cancer diagnosis has limitations in detecting cancerous lesions. Targeted prostate biopsy using multiparametric MRI/ultrasound (mpMRI/US) fusion-guided technology has become a novel standard for tissue diagnosis [5].

Globally, prostate cancer is a common and possibly fatal illness that affects many people. Efficient and precise identification is essential for successful therapy and enhanced patient results. Prostate cancer diagnosis can be improved by using deep learning models such as VGG16, VGG19, ResNet50, and ResNet50V2, which have demonstrated promise in the analysis of medical pictures. There are obstacles in implementing these algorithms in clinical practice for prostate cancer screening. Thorough comparison analyses of these architectures are required to ascertain their relative merits and drawbacks regarding prostate cancer detection accuracy. More standardization in evaluating algorithm efficacy is needed to ensure the creation of trustworthy diagnostic instruments. Furthermore, it is still imperative that deep learning models be interpretable for medical practitioners to have confidence in and comprehension of diagnostic results. Effective training and validation of machine learning models is further complicated by the dearth of comprehensive, well-documented datasets about prostate cancer. To overcome these difficulties, a comprehensive comparison of the ResNet50, VGG16, VGG19, and ResNet50V2 algorithms for prostate cancer diagnosis is being carried out in this work. By assessing the model's performance, interpretability, and generalization capacities, the research hopes to shed light on how best to use machine learning to create more dependable diagnostic instruments for prostate cancer diagnosis.

The main contributions of this research are as follows:

- The investigation seeks to conduct a thorough comparative analysis of prominent deep learning architectures, including VGG16, VGG19, ResNet50, and ResNet50V2, in the context of prostate cancer diagnosis. The study examines the performance and effectiveness of several models in discriminating between healthy and malignant prostate tissues. This evaluation is done using a dataset consisting of 738 healthy prostate and 3,514 infected prostate MRI images.
- The study evaluates the ability of machine learning algorithms to generalize and remain effective across various patient groups and clinical circumstances, while also assessing their robustness. Through the utilization of a vast and varied dataset, the study improves our comprehension of the algorithms' efficacy in practical scenarios, therefore providing valuable insights for clinical decision-making and diagnostic methods.
- The research provides the progress of diagnostic tools and procedures in the field of oncology by applying machine learning algorithms to prostate cancer diagnosis. The study intends to enhance the precision, effectiveness, and availability of prostate cancer diagnosis by utilizing advanced deep-learning models and a large collection of MRI images. This will ultimately result in improved patient outcomes and treatment approaches.

## 2. Literature Review

A significant aggregate of work has been done both in the domain of prostate cancer and technology (computer vision, artificial intelligence, and machine learning). Research on cancer detection, commonly, has gained a lot of adhesion in the recent past. Research was conducted using different types of artifacts such as mpMRI scans, CT scans, and histopathological slides to understand and detect cancer better. The contemporary inspection is focused on evaluating various techniques and models to establish efficient identification and classification systems for prostate cancer detection. Previous investigations applying ML techniques to classify and detect prostate cancer have been conducted. Khan [6] detected prostate cancer using several machine learning algorithm models (conventional machine learning, Bernoulli Naive Bayesian, passive aggressive-K-nearest neighbors, random forest, support vector, logistic regression, linear and quadratic discriminant analysis, convolutional neural networks, vanilla model and variations, and XmasNet). Overall accuracy obtained was 36–40%. The paper only considers a small number of datasets comprised of mpMRI scans of 99 patients. The project was carried out in collaboration with the Helsinki University Hospital. Yoo et al. [7] proposed deep convolutional neural networks used for the detection of prostate cancer. In this work, they built a two-step automated deep-learning pipeline for slice-level and patient-level PCa diagnosis using DWI images. A stack of five CNNs was used to produce improved classification results at the slice-level. Their best CNN (CNN1) achieved the DWI slice-level AUC of 0.87. The patient-level AUC by our random forest classifier with the features extracted through CNNs was 0.84. The paper does not consider any other model that can use only CNNs. Mehralivand et al. [8] focused on magnetic resonance imaging deep learning-based artificial intelligence for prostate cancer detection. This paper was performed to present a fully automated DL-based prostate cancer detection system for prostate MRI. They present the outcomes of a DL-based cascaded fully automated detection model for prostate cancer on parametric MRI using two different architectures. This paper adopted a 3D UNet model and an AH-Net model. Overall patient-level cancer detection sensitivity was similar between the two models, with 92.2% and 95.3% sensitivity for UNet and AH-Net models, respectively. This retrospective study includes 525 patients from two different institutions who underwent multiparametric prostate MRI and subsequent MRI-targeted prostate biopsy for clinical suspicion of prostate cancer. The goal of Tejaswi [9] was to investigate the possibility that the information contained in the cell images alone could predict the cancer risk while completely disregarding the information about how the cells are positioned in the tissue. In this project, a deep learning-based image segmentation method was used to segment cell images from the WSIs. The segmented cell images were then used for training a MIL model. Basically, this work and model only was used for the low-risk vs. high-risk prediction and in determining the cancer risk, but they alone are not the best at determining the risk. Gummeson [10] proposes a method to implement a classifier for microscopic images of potentially cancerous tissues from prostates using a convolutional neural network. There was an error rate of 7.3%. This paper does not add more classes, a more segmentation-oriented method might be interesting to use. Patel et al. [11] evaluate a number of datasets to detect prostate cancer using a deep learning framework. In research, it is observed that robust ML methodologies for classification such as SVM kernel, decision tree, and the Bayesian approach are used for separating cancer cells from the subject such as brachytherapy. It contains a total of 158 patients, among which 96 are train case patients and 62 are test cases. The model of the 3D CNN was used in these datasets and finally, they obtained the confusion matrix with an accuracy of 0.82, precision of 0.86, as well as recall of 0.78 on the validation set. In research, only the model of the 3D CNN was used in these datasets. Hosseinzadeh et al. [12] developed DL–DL-assisted prostate cancer detection on biparametric MRI: minimum training data size requirements and effect of prior knowledge. They demonstrate that the performance of a DL-CAD system for the detection and localization of csPCa in biopsy-naive men is improved by using prior knowledge of DL-based zonal segmentation. The DL sensitivity for detecting PI-RADS 4 lesions was 87% at an average of 1 false positive (FP) per patient, and an AUC of 0.88. The DL sensitivity for the detection of Gleason 6 lesions was 85% for a consensus panel of expert radiologists, if their AI needs substantially more than 2,000 training cases to achieve expert performance. Nematollahi et al. [13] proposed a review paper. Basically, they review some supervised machine-learning methods and their comparison to each other. In research, they inquire some supervised machine-learning methods' (SVM, k-nearest neighbors, decision tree, random forest, and Naive Bayes) accuracy and AUC obtained stand in (74%–92%, 0.66–0.93). Alkadi et al. [14] proposed a DL-based approach for the detection and localization of prostate cancer in T2 magnetic resonance images. They address the problem of prostate lesion detection, localization, and segmentation in T2W magnetic resonance (MR) images. The system achieves an accuracy of 0.894. This paper only considers a limited number of datasets and a simple yet efficient, deep learning-based approach for joint prostate segmentation. Almost all of these investigations have been done with a certain number of datasets and certain area-based datasets. To improve mental health evaluation through machine learning [15], this study used a “stacking-classifier-ensemble-learning” technique. As the meta-model, SVM combines several base models, such as neural networks and decision trees. The model obtains an impressive accuracy rate of around 98% by addressing class imbalances with SMOTE. It has utilized the concept of ensemble which we have used in our research. In our research, we collect datasets from various sources and datasets containing both affected and unaffected prostate cancer. Some papers have worked with specific models and algorithms with lower accuracy. On the other side, in our research, we developed multimodel and got better accuracy. In addition, some of the papers worked multi multimodel but they could not get better accuracy than ours. Intelligent computer programs, particularly artificial neural networks (ANNs), are increasingly used in medical informatics to aid in the early detection of prostate cancer from benign hyperplasia of the prostate. These AI algorithms have been integrated into medical devices and are widely accepted in medical applications, with over 500 academic publications per year featuring ANNs in the last decade [16]. A hybrid classifier utilizing particle swarm optimization (PSO) and the neural network method is proposed for supporting the diagnosis of prostate cancer, achieving a high diagnosis accuracy of 98% [17]. In our research, we utilized balanced data, but at the same time other papers utilized imbalanced data. Nearly, all of this research employs conventional machine learning algorithms, such as SVM, NB, KNN, and DT. This study is based on feature extraction, and feature reduction strategy using deep learning approaches for feature extraction such as VGG16, vGG19, ResNet50, and ResNet50v2, where we have also performed a feature reduction procedure to obtain optimal features using an autoencoder. We used recent classification techniques that include SVM and random forest (RF). The learning capability of the RF structure is quite high. To address some of the limitations and difficulties identified in previous studies, further research is still required. The proposed model overcomes these limitations by combining RF classification with glove embedding.

## 3. The Design Methods and Procedures

Prostate cancer is a highly common kind of cancer that affects males globally, leading to major consequences in terms of illness and death. Prompt identification and precise diagnosis are crucial for optimal treatment strategizing and enhanced patient results. Machine learning algorithms have been increasingly influential in medical image analysis, providing the opportunity to improve prostate cancer detection by automatically and objectively evaluating imaging data.

This section presents a new method for detecting prostate cancer using machine learning methods. The method focuses on extracting features from VGG16, VGG19, ResNet50, and ResNet50V2 architectures, and then classifying the data using the random forest algorithm combining deep learning models for feature extraction with a reliable ensemble learning method such as random forest shows potential for enhancing diagnostic accuracy and efficiency [18].

This research aims to examine the effectiveness of these machine learning algorithms in identifying distinguishing characteristics from prostate imaging data and precisely categorizing instances as either malignant or noncancerous. This study aims to develop a reliable and interpretable diagnostic tool for detecting prostate cancer by utilizing the advanced capabilities of deep learning architectures to capture complex patterns and structures in medical images, along with the versatility and robustness of random forest classification [19].

This research aims to tackle crucial issues in prostate cancer diagnosis, such as interpretability, generalization, and scalability, by thoroughly examining design methodologies and procedures, which encompass dataset preparation, model training, validation, and evaluation. The suggested methodology seeks to establish a connection between sophisticated machine learning techniques and clinical practice and to facilitate more effective and precise identification of prostate cancer. This, in turn, will result in enhanced patient care and improved results.

### 3.1. System Design

**Figure 1:**
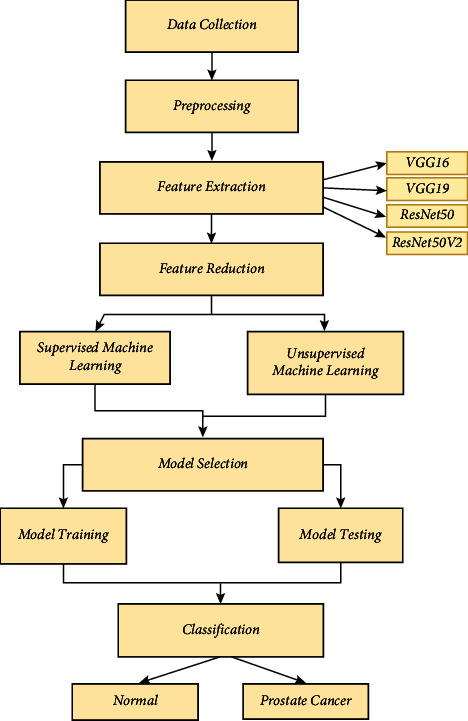
Prostate cancer detection methodology.

The focus of this study is to create machine learning-based prostate cancer detection. The proposed method has four parts: (i) corpus creation, (ii) feature extraction, (iii) feature reduction, and (iv) classification by the stack model and the methodology is shown in Figure 1.

#### 3.1.1. Corpus Creation

The process of creating a corpus for detecting prostate cancer using machine learning techniques, including VGG16, VGG19, ResNet50, and ResNet50V2, entails several important procedures to guarantee the production of a diverse and high-quality dataset suitable for training and evaluating the models.

**Figure 2:**
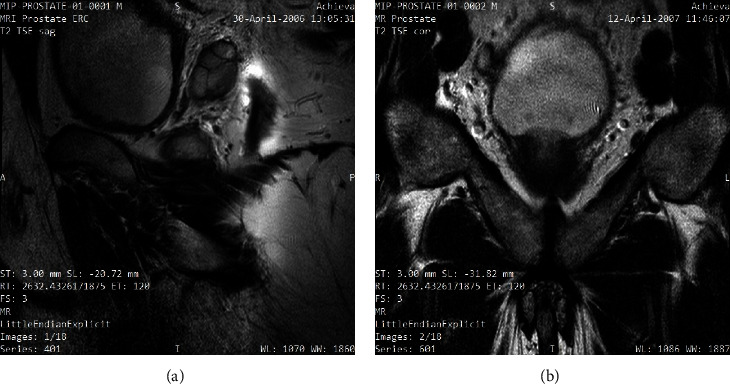
Sample MRI images of: (a) healthy prostate and (b) infected prostate.

(i) Data collection includes the following procedure:
  - MRIs of the prostate were taken with a 3T endorectal and phased array surface coil (Philips Achieva). Each patient got a robotic-assisted radical prostatectomy after biopsy confirmation of malignancy. From each MRI, a mold was made, and the prostatectomy specimen was inserted in it and sliced in the same plane. The 2008–2010 statistics came from the National Cancer Institute in Bethesda, Maryland [20].
  - Engage in partnerships with medical institutes or databases to obtain annotated MRI datasets that include accurate labels showing the presence or absence of prostate cancer.
  - Be careful in incorporating photos from various patients, imaging devices, and imaging protocols to encompass the diversity in imaging situations.
  - The size of our dataset is 22,036 MRI pictures. We have chosen a subset of 4,252 MRI scans for our investigation. Out of the total number of photos, 738 images are associated with healthy prostate samples, whereas 3,514 images represent cases of infected prostate. The selection of this particular group was made to guarantee the inclusion of both healthy and infected prostate states, hence enabling a thorough investigation within the parameters of our research. In Figure 2, sample healthy and infected prostate MRI images are shown.
(ii) Data preprocessing includes the following procedure:

- Normalize the format and resolution of MRI images to guarantee uniformity throughout the collection
- Apply picture normalization and intensity normalizing techniques to improve the comparability of images
- Utilize preprocessing methods, such as cropping, resizing, and noise reduction, to improve the quality of MRI images and concentrate on the pertinent regions of interest, specifically the prostate gland [21]

A summary of the collected data after preprocessing is shown in Table 1.

#### 3.1.2. Feature Extraction

Feature extraction is crucial in the identification of prostate cancer using machine learning algorithms such as VGG16, VGG19, ResNet50, and ResNet50V2. This procedure entails converting unprocessed MRI images into significant depictions of prominent characteristics that capture pertinent patterns and structures indicative of prostate cancer. The subsequent section delineates the approach for extracting features [22].

(i) Input preparation includes the following procedure:
  - Import the MRI image collection that includes both malignant and benign prostate images.
  - Standardize the pixel values to a uniform range (e.g., [0, 1]) to enhance the convergence and stability of the model during training.
  - Original MRI images were various in sizes as mentioned in Table 1. Adjust the size of the photos to correspond with the specified dimensions required by the pretrained deep learning models, such as 224 × 224 pixels. As we are utilizing the pretrain VGG and ResNet model, resizing to the 224 × 224 is necessary.
(ii) Feature extraction using pretrained models includes the following:
  - Employ the convolutional layers of pretrained VGG16, VGG19, ResNet50, and ResNet50V2 models as feature extractors
  - Eliminate the fully connected layers, namely the top layers, from the pretrained models, while keeping only the convolutional basis
  - Pass each MRI picture through the convolutional base of the models to extract hierarchical characteristics at various levels of abstraction
(iii) Feature pooling includes the following:
  - Utilize spatial pooling methods, such as global average pooling, to combine the derived information across spatial dimensions
  - Pooling decreases the number of dimensions in the feature maps while retaining the most distinguishing information
(iv) Feature normalization includes the following procedure:
  - Standardize the pooled vectors of features to ensure uniform scaling and reduce the influence of variances in feature magnitude across multiple layers and images
  - To improve model convergence and generalization, it is necessary to standardize the feature vectors by adjusting them to have a mean of zero and a variance of one
(v) Feature concatenation includes the following procedure:
  - Combine the aggregated feature vectors obtained from several layers of the convolutional base to form a unified feature representation
  - Through employing this fusion technique, the model can effectively capture both low-level and high-level characteristics, hence improving the discriminative ability of the retrieved features.
(vi) Output representation includes the following procedure:
  - The computer feature vectors for every MRI image in the collection, encapsulating the acquired attributes that are pertinent to the identification of prostate cancer
  - Organize the feature vectors and their related labels (identifying malignant or noncancerous) together to create input-output pairs for future classification tasks

Following this feature extraction process, the MRI image dataset transforms a collection of useful feature vectors encapsulating crucial properties for identifying prostate cancer. The collected features are used as input for the classification algorithms, enabling a precise and efficient diagnosis of prostate cancer [9].

#### 3.1.3. Logistic Regression

**Figure 3:**
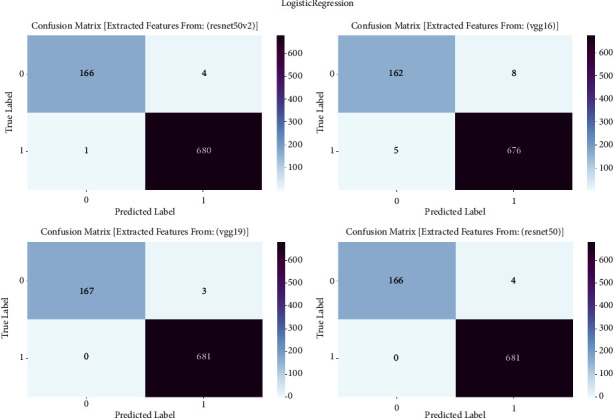
Confusion matrix of logistic regression using all features.

The confusion matrix is an essential tool for assessing the efficacy of machine learning algorithms in detecting prostate cancer. This study report used the confusion matrix to assess the classification outcomes of logistic regression models trained on features derived by VGG16, VGG19, ResNet50, and ResNet50V2 architectures. This matrix presents a detailed analysis of true positive, true negative, false positive, and false negative predictions. It allows us to evaluate the accuracy, sensitivity, specificity, and overall performance of the models in differentiating between cancerous and noncancerous prostate MRI images [7]. The confusion matrix is showng in Figure 3 and result summary in Table 2.

Logistic regression consistently exhibits robust performance across all models, with accuracy levels ranging from 98.48% to 99.64%. VGG19 achieves the maximum accuracy of 99.64%, followed closely by ResNet50 and ResNet50V2 with accuracies of 99.52% and 99.41%, respectively. The F1 scores, which measure the balanced average of precision and recall, routinely surpass 98.47%, showing a strong performance in the classification of prostate cancer. Furthermore, the recall values span from 97.28% to 99.11%, indicating the models' proficiency in accurately detecting genuine positive cases. The precision scores range from 98.47% to 99.64%, indicating that the models have a strong ability to decrease incorrect positive predictions and consistently achieve high accuracy in identifying prostate cancer.

#### 3.1.4. Decision Tree Classifier

**Figure 4:**
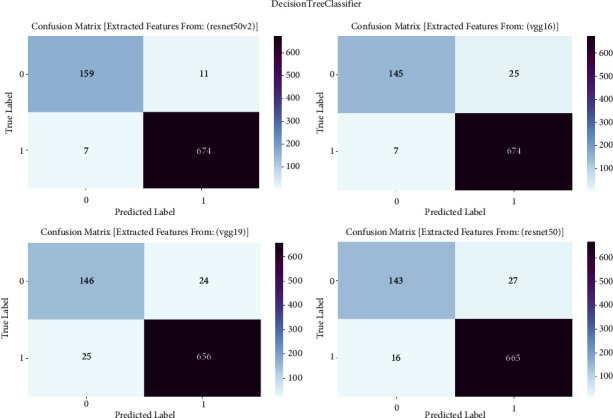
Confusion matrix of decision tree classifier (all features).

The confusion matrix is an essential evaluation tool for measuring the effectiveness of decision tree classifier models in detecting prostate cancer. This research utilizes the confusion matrix to assess classification outcomes produced by a decision tree classifier that is trained on features retrieved by the VGG16, VGG19, ResNet50, and ResNet50V2 architectures. The confusion matrix allows us to assess the accuracy, sensitivity, specificity, and overall effectiveness of the models in differentiating between cancerous and noncancerous prostate MRI images by presenting a comprehensive breakdown of true positive, true negative, false positive, and false negative predictions. The confusion matrix is shown in Figure 4 and result summary in Table 3.

The decision tree classifier demonstrates diverse performance across multiple models, with accuracy levels ranging from 94.24% to 97.89%. It is worth mentioning that ResNet50V2 demonstrates the highest level of accuracy, reaching 97.89%, which indicates its efficiency in detecting prostate cancer. The F1 scores, which measure the balance between precision and recall, range from 94.24% to 97.88%, showing a well-balanced performance in the classification of prostate cancer. Furthermore, the recall values span from 90.89% to 96.26%, indicating the models' proficiency in accurately detecting true positive cases. The precision scores vary from 94.87% to 97.88%, indicating that the models are highly effective in reducing false positive predictions and achieving accurate classification of prostate cancer.

#### 3.1.5. Gaussian NB

**Figure 5:**
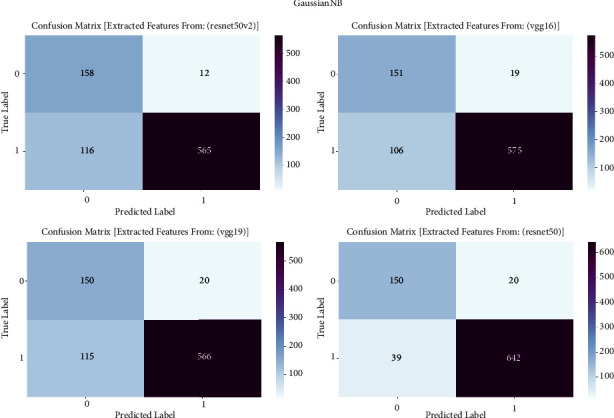
Confusion matrix of Gaussian NB (all features).

The confusion matrix is an essential evaluation technique used to examine the performance of Gaussian NB models in our study on prostate cancer diagnosis. The confusion matrix is employed to examine the classification outcomes achieved by Gaussian NB, which is trained on features derived from VGG16, VGG19, ResNet50, and ResNet50V2 architectures. The confusion matrix offers a comprehensive analysis of the accuracy, sensitivity, specificity, and overall effectiveness of models in differentiating between cancerous and noncancerous prostate MRI images. It achieves this by providing a thorough breakdown of true positive, true negative, false positive, and false negative predictions. The confusion matrix is shown in Figure 5 and result summary in Table 4.

The performance of Gaussian NB varies among different models, with accuracy ranging from 84.13% to 93.07%. The ResNet50 model attains a remarkable accuracy of 93.07%, demonstrating its efficacy in detecting prostate cancer. The F1 scores, which measure the balance between precision and recall, vary from 85.28% to 93.20%, demonstrating a well-balanced performance in the classification of prostate cancer. Furthermore, the recall values span from 85.68% to 91.25%, indicating the models' proficiency in accurately detecting genuine positive cases. The precision scores vary from 88.60% to 93.47%, indicating that the models can minimize incorrect positive predictions and retain a high level of accuracy in identifying prostate cancer.

#### 3.1.6. K-Neighbors Classifier

**Figure 6:**
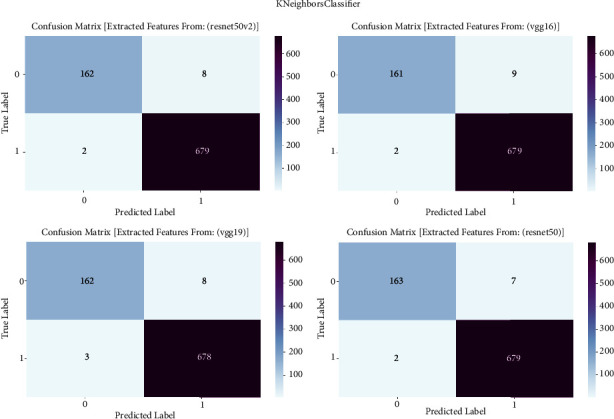
Confusion matrix of K-neighbors classifier (all features).

The confusion matrix is an essential evaluation instrument in our study on prostate cancer diagnosis. It allows us to examine the performance of K-neighbors classifier models. To investigate the classification outcomes produced by K-neighbors classifier, we utilize the confusion matrix. The classifier is trained using features taken from the VGG16, VGG19, ResNet50, and ResNet50V2 architectures. The confusion matrix provides a thorough assessment of the accuracy, sensitivity, specificity, and overall effectiveness of the models in distinguishing between cancerous and noncancerous prostate MRI images. It achieves this by presenting a detailed breakdown of true positive, true negative, false positive, and false negative predictions. The confusion matrix is shown in Figure 6 and result summary in Table 5.

The K-neighbors classifier consistently exhibits good performance across all models, with accuracy levels ranging from 98.70% to 98.94%. ResNet50 has exceptional accuracy, reaching a remarkable 98.94%, which highlights its efficacy in detecting prostate cancer. The F1 scores, which represent the balanced average of precision and recall, routinely surpass 98.70%, demonstrating the strong performance of the model in accurately classifying prostate cancer. Furthermore, the recall values vary between 97.20% and 97.80%, indicating the models' proficiency in accurately detecting true positive cases. The precision scores continuously range from 98.70% to 98.94%, indicating that the models are highly effective in minimizing false positive predictions and maintaining a high level of precision when categorizing prostate cancer.

#### 3.1.7. Linear Discriminant Analysis

**Figure 7:**
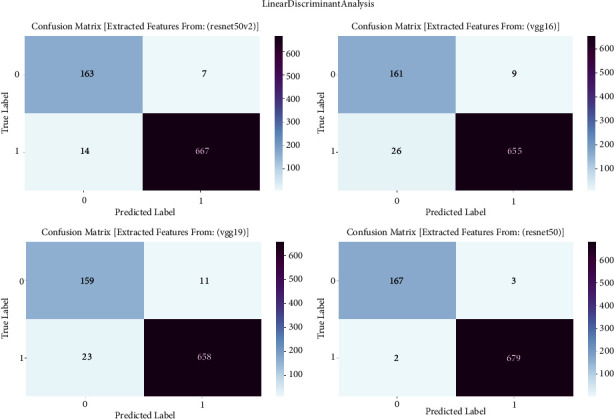
Confusion matrix of linear discriminant analysis (all features).

The confusion matrix is of utmost importance in our study on prostate cancer detection since it serves as a critical tool for evaluating the performance of linear discriminant analysis (LDA) models. The confusion matrix is employed to examine the classification results obtained by LDA, which is trained on features derived from VGG16, VGG19, ResNet50, and ResNet50V2 architectures. The confusion matrix offers a thorough evaluation of the accuracy, sensitivity, specificity, and overall efficacy of models in distinguishing between cancerous and noncancerous prostate MRI images. It achieves this by providing a detailed breakdown of true positive, true negative, false positive, and false negative predictions. The confusion matrix is shown in Figure 7 and result summary in Table 6.

The linear discriminant analysis (LDA) consistently achieves high accuracy across all models, with accuracy rates ranging from 95.89% to 99.41%. ResNet50 demonstrates exceptional efficacy in prostate cancer detection, attaining a remarkable accuracy rate of 99.41%. The F1 scores, which represent the balanced average of precision and recall, routinely surpass 95.96%, showing a strong and reliable performance of the model in accurately classifying prostate cancer. Furthermore, the recall values vary between 95.07% and 98.98%, indicating the models' proficiency in accurately detecting true positive cases. The precision scores continuously range from 96.13% to 99.41%, indicating that the models are highly effective in minimizing false positive predictions and achieving accurate classification of prostate cancer with high precision.

#### 3.1.8. SVC

**Figure 8:**
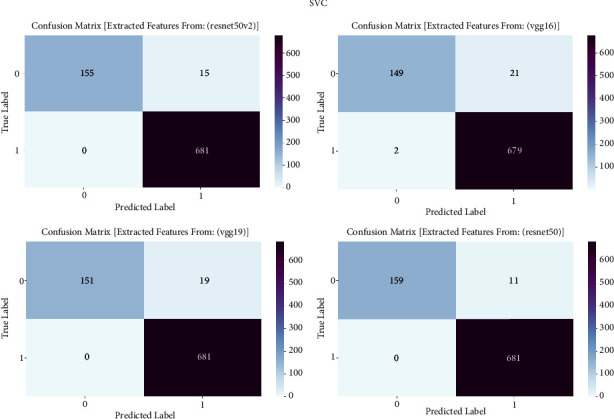
Confusion matrix of SVC (all features).

The confusion matrix is an essential evaluation tool in our study on prostate cancer diagnosis, specifically for measuring the performance of support vector classifier (SVC) models. The confusion matrix is employed to examine the classification results achieved by the support vector classifier (SVC) trained on features taken from the VGG16, VGG19, ResNet50, and ResNet50V2 architectures. The confusion matrix offers a thorough evaluation of the accuracy, sensitivity, specificity, and overall effectiveness of models in distinguishing between cancerous and noncancerous prostate MRI images. It achieves this by providing a detailed breakdown of true positive, true negative, false positive, and false negative predictions. The confusion matrix is shown in Figure 8 and result summary in Table 7.

The support vector classifier (SVC) exhibits strong performance across all models, with accuracy levels ranging from 97.30% to 98.70%. It is worth mentioning that ResNet50 achieves the highest level of accuracy, specifically 98.70%, while ResNet50V2 comes in a close second with an accuracy of 98.23%. The F1 scores, which indicate the balanced average of precision and recall, routinely surpass 97.23%, demonstrating the strong performance of the model in accurately classifying prostate cancer. Furthermore, the recall values span from 93.68% to 96.77%, indicating the models' proficiency in accurately detecting genuine positive cases. The precision scores continuously range from 97.33% to 98.72%, indicating that the models are highly effective at reducing false positive predictions and achieving accurate classification of prostate cancer.

#### 3.1.9. Stacking Classifier

**Figure 9:**
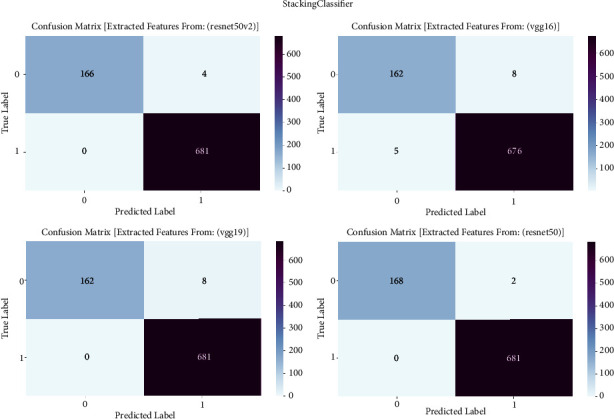
Confusion matrix of stacking classifier (all features).

The confusion matrix is an essential evaluation tool in our study on prostate cancer diagnosis. It is used to examine the performance of the stacking classifier ensemble learning technique. The confusion matrix is employed to examine the classification results obtained by the stacking classifier. This classifier mixes predictions from many base classifiers that are trained on features collected from VGG16, VGG19, ResNet50, and ResNet50V2 architectures. The confusion matrix provides a thorough assessment of the accuracy, sensitivity, specificity, and overall effectiveness of the stacking classifier in differentiating between cancerous and noncancerous prostate MRI images. It achieves this by presenting a detailed breakdown of true positive, true negative, false positive, and false negative predictions. The confusion matrix is shown in Figure 9 and result summary in Table 8.

The stacking classifier has exceptional performance across all models, with accuracy levels ranging from 98.48% to 99.77%. It is worth mentioning that ResNet50 obtains the highest level of accuracy, reaching 99.77%. Following closely behind is ResNet50V2 with an accuracy of 99.52%. The F1 scores, which represent the balanced average of precision and recall, frequently surpass 98.47%, suggesting a strong and reliable performance of the model in accurately classifying prostate cancer. Furthermore, the recall values vary between 97.28% and 99.41%, indicating the models' proficiency in accurately detecting true positive cases. The precision scores consistently range from 98.47% to 99.77%, indicating that the models are highly effective in minimizing false positive predictions and accurately classifying prostate cancer with a high level of precision.

### 3.2. Feature Reduction

Feature reduction is an essential element in the process of prostate cancer diagnosis utilizing machine learning algorithms, specifically random forest [23]. This approach prioritizes the identification and selection of the most informative characteristics from the dataset of MRI images, resulting in enhanced efficiency and interpretability of the model. The subsequent steps delineate the process of feature reduction:

(i) Feature extraction includes the following procedure:
  - Utilize approaches such as manual feature extraction or pretrained convolutional neural networks (CNNs) such as VGG16, VGG19, ResNet50, or ResNet50V2 to obtain a complete range of features from the MRI images
  - These parameters include different characteristics of the prostate gland's shape, structure, and brightness, which might detect possible signs of malignant tissue
(ii) Feature representation includes the following procedure:
  - Generate each MRI image in the dataset into a feature vector, where each member represents a single extracted feature
  - Standardize and normalize the feature vectors to ensure consistency and aid in model convergence
(iii) Feature selection includes the following procedure:
  - Utilize approaches such as filter approaches involve evaluating the relevance of features using statistical metrics such as correlation, mutual information, or chi-squared tests.
  - Wrapper approaches involve the iterative evaluation of feature subsets using a predictive model. The goal is to select the subset that maximizes performance, for example, through recursive feature elimination.
  - Embedded approaches utilize the random forest classifier's inherent ability to assess the relevance of features, allowing for the ranking of features based on their contribution to classification.
(iv) Random forest classifier training includes the following procedure:
  - Develop a random forest classifier using the reduced set of features to differentiate between prostate photos that include cancer and those that do not
  - The collective learning aspect of random forest enables it to easily handle high-dimensional data and deliver strong classification performance
(v) Evaluation and validation include the following procedure:
  - Analyze the effectiveness of the random forest classifier by using measures such as accuracy, sensitivity, specificity, and the region under the receiver operating characteristic (ROC) curve
  - Evaluate the efficacy of feature reduction by comparing the classifier's performance when utilizing the smaller feature set versus the whole feature set
  - Conduct cross-validation or holdout validation to evaluate the model's capacity to generalize and provide consistent and trustworthy findings across various subsets of the dataset.

This methodology requires improving the efficiency, interpretability, and predictive accuracy of the prostate cancer detection model by using feature reduction techniques in combination with random forest classification. The goal is to provide more precise and dependable diagnoses based on MRI image data.

#### 3.2.1. Logistic Regression

**Figure 10:**
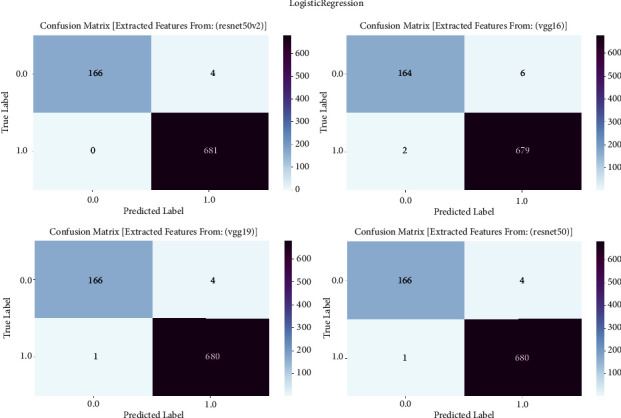
Confusion matrix of logistic regression (reduced features).

The confusion matrix is an essential tool in our research on the prostate cancer diagnosis. It is used to evaluate the performance of logistic regression models after reducing the number of features. We employ the confusion matrix to examine the classification results obtained from logistic regression models trained on feature sets taken from VGG16, VGG19, ResNet50, and ResNet50V2 architectures, after reducing the number of features. The confusion matrix allows for a thorough evaluation of the effectiveness of feature reduction techniques in enhancing the accuracy, sensitivity, specificity, and overall performance of logistic regression models. It achieves this by providing a detailed breakdown of true positive, true negative, false positive, and false negative predictions. The confusion matrix after feature reduction is shown in Figure 10 and result summary in Table 9.

Logistic regression consistently achieves outstanding performance across all models, exhibiting accuracy levels ranging from 99.05% to 99.52%. ResNet50 and ResNet50V2 attain the utmost precision with accuracy rates of 99.41% and 99.52% correspondingly. The F1 scores, which measure the balanced average of precision and recall, routinely surpass 99.05%, demonstrating strong performance in the classification of prostate cancer. The recall values vary between 98.09% and 98.82%, indicating the models' capacity to accurately detect true positive cases. The precision scores regularly range from 99.05% to 99.53%, indicating that the models have a strong ability to decrease false positive predictions and retain high precision when identifying prostate cancer.

#### 3.2.2. Decision Tree Classifier

**Figure 11:**
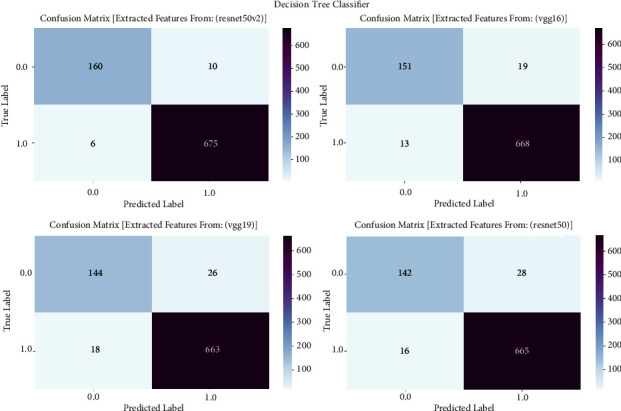
Confusion matrix of decision tree classifier (reduced features).

During our research on the prostate cancer diagnosis, the confusion matrix plays a crucial role in assessing the effectiveness of feature reduction approaches when used alongside the decision tree classifier algorithm. We employ the confusion matrix to examine the classification results of decision tree classifier models that were trained on feature sets taken from VGG16, VGG19, ResNet50, and ResNet50V2 architectures, after reducing the number of features. The confusion matrix allows for a thorough evaluation of the effects of feature reduction on the accuracy, sensitivity, specificity, and overall performance of decision tree classifier models by presenting a detailed breakdown of true positive, true negative, false positive, and false negative predictions. The confusion matrix after feature reduction is shown in Figure 11.

The decision tree classifier demonstrates variable performance across multiple models, with accuracy levels ranging from 94.82% to 98.11%. The ResNet50V2 model achieves the highest accuracy rate of 98.11%, while the VGG16 model follows closely with an accuracy rate of 96.23%. The F1 scores, which measure the balanced performance of identifying prostate cancer by considering both precision and recall, vary from 94.76% to 98.11%. The recall scores vary between 90.59% and 96.61%, indicating the models' proficiency in accurately detecting true positive cases. The precision scores continuously range from 94.74% to 98.10%, indicating that the models are highly effective at reducing false positive predictions and accurately classifying prostate cancer with a high level of precision.

#### 3.2.3. Gaussian NB

**Figure 12:**
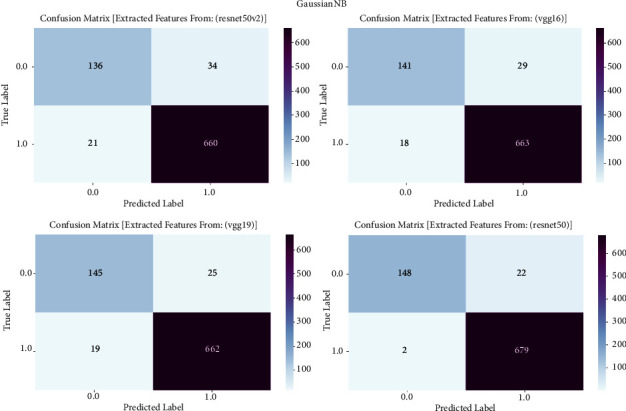
Confusion matrix of Gaussian NB (reduced features).

During our research on prostate cancer detection, we utilize the confusion matrix as a crucial tool to assess the efficacy of feature reduction strategies when combined with the Gaussian NB algorithm. We utilize the confusion matrix to analyze the classification results of Gaussian NB models that were trained on feature sets taken from VGG16, VGG19, ResNet50, and ResNet50V2 architectures, after reducing the number of features. The confusion matrix provides a comprehensive evaluation of the impact of feature reduction on the accuracy, sensitivity, specificity, and overall performance of Gaussian NB models in detecting prostate cancer. It achieves this by providing a detailed breakdown of true positive, true negative, false positive, and false negative predictions. The confusion matrix after feature reduction is shown in Figure 12.

The Gaussian NB model consistently achieves strong performance across all models, with accuracy levels ranging from 93.53% to 97.18%. The accuracy achieved by ResNet50 is 97.18%, which is the greatest among all models. VGG19 follows with an accuracy of 94.82%. The F1 scores, which measure the balance between precision and recall, vary from 93.40% to 97.11%. This indicates that the classification of prostate cancer shows a balanced performance. The recall scores vary between 88.46% and 93.39%, indicating the models' proficiency in accurately detecting true positive cases. The precision scores continuously range from 93.40% to 97.22%, indicating that the models are highly effective in reducing false positive predictions and achieving accurate classification of prostate cancer.

#### 3.2.4. K-Neighbors Classifier

**Figure 13:**
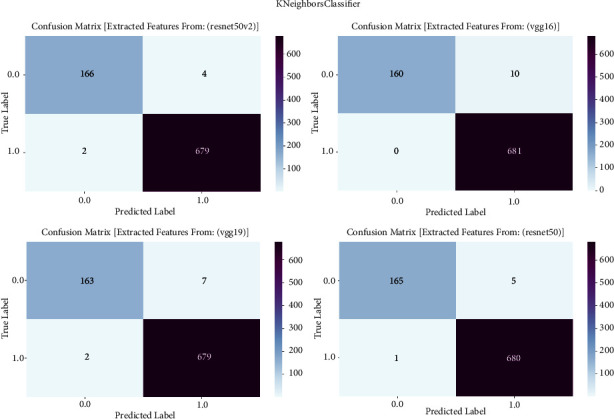
Confusion matrix of K-neighbors classifier (reduced features).

The confusion matrix is an essential tool in our study on prostate cancer diagnosis. It allows us to evaluate the effectiveness of feature reduction approaches when used in combination with the K-neighbors classifier algorithm. We employ the confusion matrix to examine the classification results of K-neighbors classifier models that were trained on feature sets taken from VGG16, VGG19, ResNet50, and ResNet50V2 architectures, after reducing the number of features. The confusion matrix offers a thorough evaluation of the impact of feature reduction on the accuracy, sensitivity, specificity, and overall performance of K-neighbors classifier models in prostate cancer detection. It achieves this by providing a detailed breakdown of true positive, true negative, false positive, and false negative predictions. The confusion matrix after feature reduction is shown in Figure 13.

The K-neighbors classifier has excellent results across all models, with accuracy levels ranging from 98.82% to 99.30%. Both ResNet50 and ResNet50V2 demonstrate exceptional accuracy, reaching a peak of 99.30%. VGG19 closely trails behind with an accuracy of 98.94%. The F1 scores, which measure the balanced performance of precision and recall using the harmonic mean, routinely surpass 98.81%, demonstrating the robustness of the model in achieving a balance between accuracy and recall. In addition, the recall values vary between 97.05% and 98.68%, indicating the models' proficiency in accurately detecting true positive cases. The precision scores consistently range from 98.84% to 99.30%, indicating that the models are highly effective in minimizing false positive predictions and maintaining a high level of precision in classifying prostate cancer.

#### 3.2.5. Linear Discriminant Analysis

**Figure 14:**
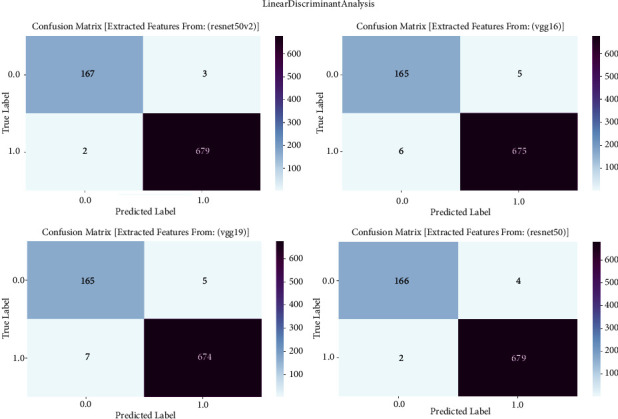
Confusion matrix of linear discriminant analysis (reduced features).

The confusion matrix is of crucial significance in evaluating the efficacy of feature reduction approaches when used in conjunction with the linear discriminant analysis (LDA) algorithm in our study on prostate cancer diagnosis. We utilize the confusion matrix to evaluate the classification results of LDA models that were trained on feature sets taken from VGG16, VGG19, ResNet50, and ResNet50V2 architectures after reducing their dimensions. The confusion matrix provides a comprehensive analysis of the impact of feature reduction on the accuracy, sensitivity, specificity, and overall performance of LDA models in detecting prostate cancer. It achieves this by providing a detailed breakdown of true positive, true negative, false positive, and false negative predictions. The confusion matrix after feature reduction is shown in Figure 14.

The linear discriminant analysis (LDA) demonstrates excellent performance across all models, achieving accuracy rates ranging from 98.59% to 99.41%. It is worth mentioning that ResNet50V2 obtains the highest level of accuracy, reaching 99.41%, while ResNet50 comes in a close second with an accuracy of 99.30%. The F1 scores, which represent the harmonic mean of precision and recall, routinely surpass 98.60%, demonstrating the strong performance of the model in achieving a balance between precision and recall. Furthermore, the recall scores vary between 98.09% and 98.98%, indicating the models' proficiency in accurately detecting true positive cases. The precision scores continuously range from 98.60% to 99.41%, indicating that the models are highly effective at minimizing false positive predictions and maintaining a high level of precision in identifying prostate cancer.

#### 3.2.6. SVC

**Figure 15:**
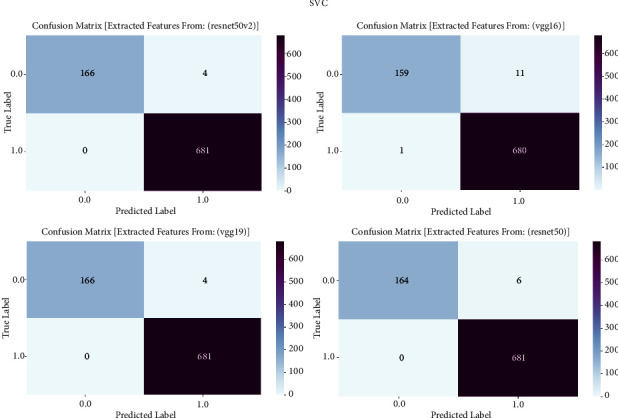
Confusion matrix of SVC (reduced features).

The confusion matrix is a crucial tool in our study on the prostate cancer diagnosis. It allows us to evaluate the effectiveness of feature reduction approaches when used with the support vector classifier (SVC) algorithm. The confusion matrix is employed to examine the classification results of support vector classifier (SVC) models trained on feature sets taken from VGG16, VGG19, ResNet50, and ResNet50V2 architectures, after reducing the number of features. The confusion matrix allows for a thorough evaluation of the impact of feature reduction on the accuracy, sensitivity, specificity, and overall performance of support vector classification (SVC) models in detecting prostate cancer. It achieves this by providing a detailed breakdown of true positive, true negative, false positive, and false negative predictions. The confusion matrix after feature reduction is shown in Figure 15.

The support vector classifier (SVC) demonstrates exceptional performance across all models, achieving accuracy levels ranging from 98.59% to 99.52%. VGG19 notably achieves the greatest accuracy rate of 99.52%, closely followed by ResNet50V2 with a rate of 99.30%. The F1 scores, which measure the balance between precision and recall using the harmonic mean, routinely surpass 98.58%, demonstrating the strong performance of the model in achieving a balance between precision and recall. Furthermore, the recall values span from 96.70% to 98.82%, demonstrating the models' capacity to accurately detect true positive cases. The precision scores constantly exhibit a high level of accuracy, ranging from 98.60% to 99.53%. This highlights the models' capacity to effectively reduce false positive predictions and uphold a high level of precision in the classification of prostate cancer.

#### 3.2.7. Stacking Classifier

**Figure 16:**
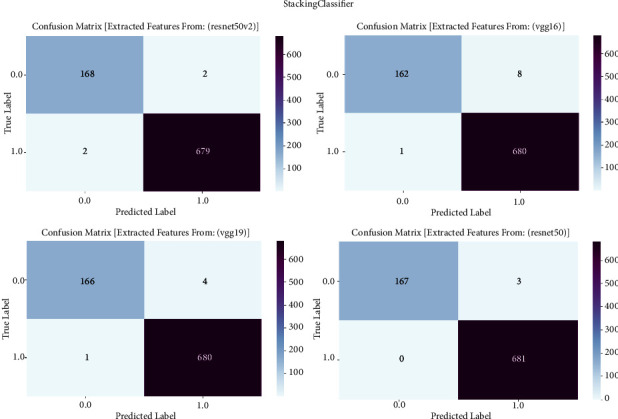
Confusion matrix of stacking classifier (reduced features).

The confusion matrix is an essential tool in our research on the prostate cancer diagnosis. It allows us to evaluate the performance of feature reduction approaches when used in conjunction with the stacking classifier ensemble learning algorithm. To examine the classification outcomes of stacking classifier models trained on reduced feature sets taken from VGG16, VGG19, ResNet50, and ResNet50V2 architectures, we utilize the confusion matrix. The confusion matrix allows for a thorough evaluation of the impact of feature reduction on the accuracy, sensitivity, specificity, and overall performance of stackingclassifier models in prostate cancer detection. It achieves this by providing a detailed breakdown of true positive, true negative, false positive, and false negative predictions. The confusion matrix after feature reduction is shown in Figure 16.

The stacking classifier demonstrates outstanding performance across all models, with a high level of accuracy ranging from 98.94% to 99.64%. It is worth mentioning that ResNet50 demonstrates the highest level of accuracy, reaching 99.64%, while ResNet50V2 closely follows with an accuracy of 99.52%. The F1 scores, which represent the harmonic mean of precision and recall, routinely exceed 98.93%, demonstrating the model's strong performance in maintaining a balance between precision and recall. Furthermore, the recall values surpass 97.58%, demonstrating the models' effective capability in identifying real positive cases. The precision scores continuously range from 98.94% to 99.64%, indicating that the models are highly effective at reducing false positive predictions and achieving accurate classification of prostate cancer.

**Figure 17:**
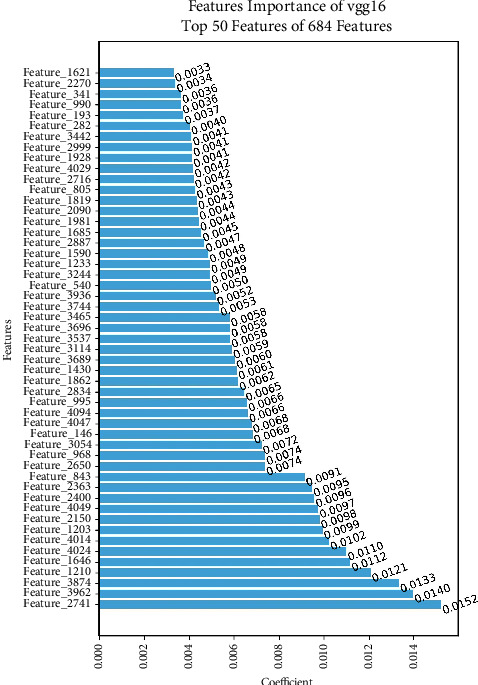
Feature importance of VGG16.

The feature importance study of prostate cancer detection using the VGG16 model identifies the top 50 features that uncover crucial patterns retrieved from MRI images, as shown in Figure 17. These traits are believed to correspond to complex textures, forms, and structures that indicate the presence of prostate cancer. The significance of these attributes resides in their capacity to capture nuanced fluctuations in image properties, facilitating precise classification. The selected top 50 features likely emphasize the importance of tumor form, tissue density, and spatial organization in differentiating between healthy and malignant prostate tissue.

**Figure 18:**
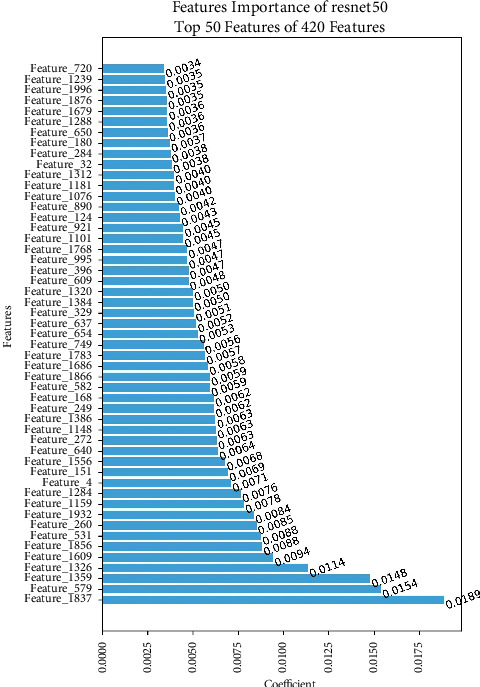
Feature importance of ResNet50.

The feature importance analysis of prostate cancer detection using the ResNet50 model reveals that the top 50 features encompass vital information collected from MRI image, as shown in Figure 18. These traits are believed to depict complex patterns, textures, and structural details that indicate the presence of prostate cancer. Notable features among those chosen include characteristics about the shape of the tumor, differences in tissue density, and spatial organization. Their importance rests in their capacity to detect nuanced distinctions between normal and malignant prostate tissue, facilitating precise categorization. The ResNet50 model's capabilities for a precise and consistent diagnosis of prostate cancer are greatly enhanced by these key elements.

### 3.3. Feature Analysis

Table 10 demonstrates the results of employing the random forest approach for feature reduction in the detection of prostate cancer utilizing machine learning techniques, namely VGG16, VGG19, ResNet50, and ResNet50V2. At first, all algorithms processed a dataset containing 4,253 extracted features. Following the reduction of features, notable decreases were observed in all algorithms, resulting in improved computational efficiency and potentially better model performance.

Logistic regression, which originally employed 4,253 features, was optimized to function well using a reduced set of 714 features. Similarly, the decision tree classifier, Gaussian Naive Bayes, K-neighbors classifier, linear discriminant analysis, support vector classifier, and stacking classifier algorithms similarly underwent significant decreases in features, with the number of features ranging from 668 to 708.

The decrease in features indicates that the random forest algorithm successfully recognized and preserved the most significant characteristics related to prostate cancer detection, while removing unnecessary or less influential ones. The concise collection of features is anticipated to enhance model performance, expedite training, and improve prediction efficiency. This underscores the effectiveness of the random forest approach in optimizing the feature space for greater prostate cancer detection utilizing different machine learning algorithms.

#### 3.3.1. Predicted Output

This research report introduces a classification framework that uses a single-stack machine learning model to differentiate between normal and prostate cancer MRI images. Our objective is to create a precise and efficient diagnostic tool for detecting prostate cancer from MRI data by utilizing convolutional neural networks and optimizing model architecture and training techniques. The findings of this study have the capacity to make a substantial contribution to the domain of prostate cancer diagnostics by aiding in the early identification of the disease and enhancing patient outcomes [12].

The proposed single-stack machine learning model generates a binary classification outcome that distinguishes between normal and prostate cancer MRI images. This outcome represents the anticipated class label for each input picture. The model categorizes each MRI picture as either “normal” or “prostate cancer” by utilizing the acquired features and patterns during the training process. Here is an illustration of the format in which the output is presented [13].

## 4. Evaluation of the Developed System

This thoroughly evaluates a prostate cancer detection system that utilizes sophisticated machine learning techniques such as VGG16, VGG19, ResNet50, and ResNet50V2. Early identification is crucial for better treatment outcomes in prostate cancer, a widespread illness that affects men globally. Our objective is to improve the precision and effectiveness of prostate cancer detection by utilizing advanced machine learning methods on medical imaging data. This section assesses the efficacy of different convolutional neural network (CNN) architectures in accurately classifying MRI images as either cancerous or benign. We evaluate the diagnostic capacity of each algorithm by analyzing parameters such as accuracy, F1 score, recall, and precision, allowing us to determine their respective strengths and limitations. The results of our study assist in choosing the most appropriate approach, considering criteria such as accuracy in categorization, computing speed, and resilience to fluctuations in MRI data. In addition, we analyze the obstacles and potential areas of future investigation to improve the system for improved clinical usability, thereby contributing to progress in the field of oncology diagnostics.

### 4.1. Data Collection

Our research on prostate cancer detection involved the utilization of the prostate MRI dataset accessible on the cancer imaging archive (TCIA) website. The dataset comprises an extensive assortment of multiparametric MRI (mpMRI) images obtained from patients diagnosed with prostate cancer. The dataset consists of pictures acquired using different MRI sequences, such as T2-weighted imaging, diffusion-weighted imaging, and dynamic contrast-enhanced imaging [24].

The data-collecting process entailed retrieving the prostate MRI dataset from the [20] and extracting pertinent MRI pictures for analysis. We prioritized the selection of MRI scans that possessed excellent resolution and imaging parameters to guarantee the precision and dependability of our machine-learning models.

The dataset comprises MRI scans obtained from a heterogeneous group of patients who have been diagnosed with prostate cancer. These images capture the broad spectrum of tumor characteristics, disease severity, and structural features. Every MRI scan is accompanied by comprehensive clinical metadata, which includes patient demographics, histopathological findings, and imaging methods. This metadata is crucial for training and evaluating models since it provides useful information. Our work employed a subset of the prostate MRI dataset that specifically included T2-weighted MRI pictures. These images are widely utilized in the field to detect and characterize prostate cancer. The provided photos were used as input data to train and evaluate our machine learning algorithms, specifically VGG16, VGG19, ResNet50, and ResNet50V2, to classify prostate cancer.

Our objective was to utilize the extensive and varied dataset from TCIA to create and assess powerful machine-learning models that can effectively identify prostate cancer from MRI images. By utilizing sophisticated imaging technologies and artificial intelligence, we may effectively leverage data to enhance the early detection and treatment of prostate cancer. This, in turn, leads to improved patient outcomes and more informed clinical decision-making.

### 4.2. Data Analysis

Our research focused on prostate cancer diagnosis using machine learning algorithms. We performed a thorough analysis of MRI datasets, which included 738 prostate scans from healthy individuals and 3,514 images from patients diagnosed with prostate cancer. The datasets were partitioned into two categories: “healthy” and “infected,” which corresponded to noncancerous and malignant prostate pictures, respectively. Below is the conducted data analysis:

- Data distribution: we examined the distribution of prostate images that were classified as healthy and diseased in the datasets to gain insights into the imbalance between the two classes. To provide a balanced representation for model training and evaluation, it was necessary to calculate the ratio of healthy and infected photos in each dataset.
- Image characteristics: We analyzed the attributes of MRI images in both datasets, encompassing resolution, pixel intensity distribution, and anatomical features. Analyzed disparities in image attributes between normal and diseased prostate images facilitated the identification of potential cancer detection biomarkers.
- Feature extraction was conducted on MRI images from both datasets using pretrained models including VGG16, VGG19, ResNet50, and ResNet50V2. This entailed collecting prominent characteristics from several convolutional layers of the networks, capturing significant patterns that are diagnostic of prostate cancer.
- Model training and evaluation: We conducted training of machine learning models, specifically VGG16, VGG19, ResNet50, and ResNet50V2, utilizing the collected features from both healthy and infected prostate datasets. Afterwards, we assessed the performance of each model using established assessment criteria including accuracy, precision, recall, and F1 score.
- Performance evaluation: We conducted a comparative analysis of various machine learning algorithms to assess their efficacy in differentiating between healthy and infected prostate pictures. The comparative research yielded valuable insights regarding the efficacy of each model in diagnosing prostate cancer based on MRI data.
- Validation and generalization: To guarantee the dependability and applicability of our results, we performed cross-validation and assessed the trained models using separate test sets. The validation approach facilitated the evaluation of the models' resilience and their capacity to extrapolate unfamiliar data.
- Limitations and future directions: We have addressed the constraints of our study, which encompass the size of the dataset, the imbalance in class distribution, and the probable presence of biases. In addition, we identified potential areas for future research, such as integrating more clinical data and investigating ensemble learning methods, to significantly improve the accuracy of prostate cancer detection models.

### 4.3. Optimization

To enhance the identification of prostate cancer through the utilization of machine learning algorithms, several methodologies can be employed to optimize datasets consisting of 738 MRI images of healthy prostates and 3,514 MRI images of infected prostates. It is essential to tackle the issue of class imbalance, which can be addressed by employing either oversampling or undersampling techniques. Techniques such as rotation and flipping are used to increase the variety of training samples, which is known as data augmentation. Optimizing hyperparameters such as learning rate and batch size for VGG16, VGG19, ResNet50, and ResNet50V2 models significantly improves their performance. Utilizing pretrained weights in transfer learning expedites the convergence process and enhances the accuracy of the model. Utilizing ensemble learning to aggregate predictions from different models improves the dependability of the results. We want to create exceptionally effective and dependable machine learning models for prompt prostate cancer detection using MRI data, utilizing these optimization strategies. The evaluation measures encompass precision, recall, accuracy, and F1 score, which serve as indicators of the model's performance.

(1) $$\begin{array}{c} \text{Precision}=\frac{\;T_{p}}{T_{p}+F_{p}}, \end{array}$$

(2) $$\begin{array}{c} \text{Recall}=\frac{\;T_{p}}{T_{p}+F_{n}}, \end{array}$$

(3) $$\begin{array}{c} \text{Accuracy}=\frac{\;T_{p}+T_{n}}{T_{p}+F_{p}+T_{n}+F_{n}}, \end{array}$$

(4) $$\begin{array}{c} F1‐\text{score}=\frac{2\times \text{Pecision}\times \text{Recall}}{\text{Pecision}+\text{Recall}}, \end{array}$$

where *T*_*p*_, *T*_*n*_, *F*_*p*_, and *F*_*n*_, respectively, represent the true positive, true negative, false positive, and false negative.

### 4.4. Analysis for Feature Extraction

The VGG16 model is utilized as a feature extractor in the detection of prostate cancer for several machine-learning techniques. Logistic regression attains a 98.47% accuracy, along with balanced F1 score, recall, and precision values. The decision tree classifier (DT classifier) has a slightly lower accuracy rate of 96.23%, but the Gaussian NB model performs less effectively with an accuracy of 85.31. In Table 11, the result analysis is showing the summary on VGG16 features all features.

The K-neighbors classifier algorithm obtains an impressive accuracy rate of 98.70%, demonstrating consistent and well-balanced performance across several criteria. The linear discriminant analysis (LDA) model yields a high accuracy of 95.89%, indicating excellent performance in terms of F1 score, recall, and precision. The support vector classifier (SVC) gets a high accuracy rate of 97.30%, demonstrating consistent performance across all criteria.

The stacking classifier demonstrates superior performance compared to previous algorithms, achieving an accuracy of 98.48% and balanced F1 score, recall, and precision values. In summary, the VGG16 model efficiently captures distinctive characteristics for the identification of prostate cancer, as machine learning methods attain exceptional precision and well-balanced classification outcomes.

The VGG19 model is utilized as a feature extractor in the detection of prostate cancer for several machine-learning techniques. Logistic regression attains a remarkable accuracy of 99.64%, accompanied by well-balanced F1 score, recall, and precision values. The decision tree classifier (DT classifier) achieves an accuracy of 94.24%, which is lower compared to the accuracy of 84.13% achieved by the Gaussian NB model, indicating the lowest performance. In Table 12, the result analysis shows the summary on VGG19 features all features.

The K-neighbors classifier algorithm obtains a commendable accuracy rate of 98.70%, demonstrating consistent and well-balanced performance across several criteria. The linear discriminant analysis (LDA) model yields a high accuracy of 96.00%, indicating excellent performance in terms of F1 score, recall, and precision. The support vector classifier (SVC) obtains a high accuracy of 97.77%, demonstrating consistent performance across all criteria.

The stacking classifier attains a 99.05% accuracy, along with balanced F1 score, recall, and precision values. In summary, the VGG19 model successfully captures relevant characteristics for the identification of prostate cancer, as machine learning techniques achieve exceptional precision and well-balanced performance in categorization.

The ResNet50 model functions as a feature extractor for diverse machine-learning methods employed in the detection of prostate cancer. Logistic regression attains a precision of 99.52%, along with balanced F1 score, recall, and precision metrics. The decision tree classifier (DT classifier) has an accuracy of 94.94%, which is lower than the accuracy of Gaussian NB, which is 93.07%. In Table 13, the result analysis shows the summary of ResNet50 features all features.

The K-neighbors classifier demonstrates exceptional accuracy, achieving a remarkable 98.94%. Furthermore, it maintains a well-balanced performance across several measures. The linear discriminant analysis (LDA) model obtains an accuracy of 99.41%, indicating excellent performance in terms of F1 score, recall, and precision. The support vector classifier (SVC) has a high accuracy rate of 98.70% and demonstrates balanced performance across all measures.

The stacking classifier attains a remarkable accuracy of 99.77% while maintaining balanced F1 score, recall, and precision values. In summary, the ResNet50 model efficiently captures distinctive characteristics for the identification of prostate cancer, as machine learning methods achieve a notable level of accuracy and well-balanced performance in classification.

The ResNet50V2 model functions as a feature extractor for diverse machine-learning methods employed in the detection of prostate cancer. Logistic regression attains a 99.41% accuracy, accompanied by balanced F1 score, recall, and precision values. The decision tree classifier (DT classifier) has a slightly lower accuracy rate of 97.88%, but the Gaussian NB model shows the lowest performance with an accuracy of 84.95%. In Table 14, the result analysis is showing the summary on ResNet50v2 features all features.

The K-neighbors classifier algorithm yields a commendable accuracy rate of 98.82%, demonstrating consistent and well-balanced performance across all evaluation metrics. The linear discriminant analysis (LDA) algorithm achieves a high accuracy of 97.53%, indicating excellent performance in terms of F1 score, recall, and precision. The support vector classifier (SVC) gets a high accuracy rate of 98.23%, demonstrating consistent performance across all criteria.

The stacking classifier attains a remarkable accuracy of 99.52% while maintaining balanced F1 score, recall, and precision values. In summary, the ResNet50V2 model efficiently captures distinctive characteristics for the identification of prostate cancer, demonstrating remarkable accuracy and well-balanced performance in classification through the utilization of machine learning methods.

#### 4.4.1. Analysis Result

The tables display the performance metrics of multiple machine-learning algorithms in detecting prostate cancer. The algorithms were evaluated using various feature extraction methods, namely VGG16, VGG19, ResNet50, and ResNet50V2. Below is a comprehensive examination of the outcomes:

- Logistic regression consistently demonstrates strong performance across all feature extraction approaches, with high levels of accuracy, F1 score, recall, and precision. It exhibits resilience in categorizing instances of prostate cancer.
- The performance of the decision tree classifier (DT classifier) varies, with accuracy levels ranging from 94.24% to 97.88%. Although generally effective, it demonstrates slightly inferior performance in comparison to logistic regression.
- Gaussian Naive Bayes (Gaussian NB) regularly achieves the lowest performance compared to the other algorithms under consideration. The data analysis exhibits reduced accuracy and precision, suggesting constraints in effectively managing the intricacy of the dataset.
- The K-nearest neighbors algorithm, specifically the K-neighbors classifier, consistently demonstrates strong performance across various feature extraction techniques, resulting in high accuracy and well-balanced metrics. It is proficient at discerning patterns within the dataset.
- Linear discriminant analysis (LDA) reliably attains high accuracy and maintains balanced performance across several criteria. It exhibits efficacy in segregating categories within the dataset.
- The support vector classifier (SVC) demonstrates excellent performance, attaining high accuracy and maintaining balanced metrics. Nevertheless, it demonstrates marginally inferior performance in comparison to logistic regression and LDA.
- The stacking classifier consistently attains high accuracy and balanced metrics across all feature extraction methods. By integrating the capabilities of various algorithms, it achieves enhanced performance.

Logistic regression, LDA, and stacking classifiers have been identified as the most successful algorithms for detecting prostate cancer using various feature extraction approaches. These algorithms demonstrate their ability to handle the dataset's complexity and effectively categorize cases.

### 4.5. Analysis for Feature Reduction

The VGG16 model exhibits robust performance across diverse machine-learning techniques in the realm of prostate cancer detection. The performance of logistic regression, decision tree classifier (DT classifier), Gaussian NB, K-neighbors classifier, linear discriminant analysis (LDA), support vector classifier (SVC), and stacking classifier was assessed using measures such as accuracy, F1 score, recall, and precision. In Table 15, the result analysis shows the summary on VGG16 reduced features. Logistic regression achieves a 98.59% accuracy, exhibiting balanced F1 score, recall, and precision values. The DT classifier demonstrates somewhat inferior performance, achieving an accuracy rate of 95.88%, whereas Gaussian NB earns an accuracy rate of 94.94%. The K-neighbors classifier and SVC models both obtain a high accuracy of 98.70%. However, the K-neighbors classifier model exhibits superior recall and precision compared to SVC. The latent Dirichlet allocation (LDA) model obtains a high accuracy rate of 98.00% while maintaining a balanced performance across all measures.

The stacking classifier demonstrates superior performance compared to other algorithms, achieving the greatest accuracy rate of 99.05% and balanced F1 score, recall, and precision values. In general, the VGG16 model functions as a robust tool for extracting important features that may be used by other machine learning methods. This greatly enhances the precision and dependability of prostate cancer detection from MRI scans.

The VGG19 model has exceptional efficacy across diverse machine learning techniques in the realm of prostate cancer detection. Logistic regression attains a remarkable accuracy of 99.41%, accompanied with well-balanced F1 score, recall, and precision values. The decision tree classifier (DT classifier) and Gaussian NB methods demonstrate relatively lower performance, achieving an accuracy rate of 94.82% compared to other techniques. In Table 16, the result analysis is showing the summary on VGG19 reduced features.

The K-neighbors classifier and linear discriminant analysis (LDA) models obtain an impressive accuracy of 98.94% and 98.59%, respectively. Both models demonstrate balanced performance across many criteria. The support vector classifier (SVC) and stacking classifier algorithms provide superior performance compared to other methods, obtaining an accuracy of 99.52% while maintaining balanced F1 score, recall, and precision values.

In general, the VGG19 model functions as a proficient tool for extracting features in different machine-learning methods, thereby enhancing the precision and dependability of prostate cancer detection from MRI scans. The VGG19 model demonstrates durability and efficacy in this task, as evidenced by its excellent accuracy and balanced performance across metrics.

The ResNet50 model exhibits robust performance across diverse machine-learning algorithms for the identification of prostate cancer. Logistic regression attains a remarkable accuracy of 99.41%, accompanied by balanced F1 score, recall, and precision values. The decision tree classifier (DT classifier) demonstrates a somewhat inferior performance, achieving an accuracy of 94.82%, compared to the Gaussian NB model which obtains an accuracy of 97.17%. In Table 17, the result analysis is showing the summary on ResNet50 reduced features.

The K-neighbors classifier, linear discriminant analysis (LDA), and support vector classifier (SVC) models reach a remarkable accuracy of 99.30%, demonstrating consistent performance across all criteria. The stacking classifier method exhibits superior performance compared to other algorithms, achieving the greatest accuracy rate of 99.64% and demonstrating balanced F1 score, recall, and precision values.

In general, the ResNet50 model is a highly efficient tool for extracting features that may be used by different machine learning algorithms. This greatly enhances the precision and dependability of prostate cancer detection using MRI scans. The ResNet50 model demonstrates durability and effectiveness in this task, as seen by its excellent accuracy and balanced performance across measures.

The ResNet50V2 model demonstrates exceptional efficacy across diverse machine-learning techniques for the identification of prostate cancer. Logistic regression attains a remarkable accuracy of 99.52%, accompanied with well-balanced F1 score, recall, and precision values. The decision tree classifier (DT classifier) obtains an accuracy of 98.11%, exhibiting marginally inferior performance in comparison to alternative techniques. In Table 18, the result analysis is showing the summary on VGG16 reduced features.

Gaussian NB demonstrates worse accuracy in comparison to other algorithms, reaching a rate of 93.53%. Nevertheless, K-neighbors classifier, linear discriminant analysis (LDA), support vector classifier (SVC), and stacking classifier exhibit exceptional accuracy of 99.30% or more, while maintaining a well-balanced performance across several measures.

In general, the ResNet50V2 model acts as a proficient tool for extracting features that are useful for different machine learning algorithms. This greatly enhances the precision and dependability of prostate cancer detection from MRI scans. The ResNet50V2 model demonstrates durability and efficacy in this task, as seen by its excellent accuracy and balanced performance across measures.

The results obtained from the four tables illustrate the efficacy of diverse machine-learning methods in detecting prostate cancer. This evaluation was conducted utilizing distinct feature extraction models, namely VGG16, VGG19, ResNet50, and ResNet50V2. The algorithms demonstrate good accuracy across all tables, showing their usefulness in differentiating between healthy and diseased prostate pictures.

Table 15 shows that stacking classifier achieve the maximum accuracy, all over 99.05%. These algorithms also attain excellent F1 scores, recall, and precision values, indicating a well-balanced performance in classification.

Table 16 shows the maximum accuracy, exceeding 99.4%, achieved by SVC and stacking classifier, as shown in Table 16. These algorithms consistently perform well in all parameters, indicating their resilience in detecting prostate cancer.

Table 17 shows that the stacking classifier achieves the best accuracy of 99.64%, with logistic regression, K-neighbors classifier, LDA, and SVC closely follow, all obtaining accuracies above 99.3%. These algorithms demonstrate equitable performance in terms of F1 score, recall, and precision.

Table 18 shows the best accuracy of 99.52% is achieved by SVC, LDA, and stacking classifier, as shown in Table 18. The decision tree classifier and K-neighbors classifier models exhibit exceptional performance, achieving accuracies beyond 98%. Nevertheless, Gaussian NB demonstrates inferior performance in comparison to other methods.

In general, the findings suggest that the stacking classifier consistently demonstrates strong performance across all feature extraction models, with SVC, logistic regression, and K-neighbors classifier following suit. These algorithms showcase the capacity for precise and dependable identification of prostate cancer through the utilization of machine learning methodologies.

### 4.6. Discussion

The study report analyses the performance variance of machine learning algorithms by comparing the first four tables with the last four tables, which employ different feature extraction approaches. Below is the analysis and comparison of the results:

(i) Algorithm performance when utilizing feature extraction includes the following:
  - The performance metrics (accuracy, F1 score, recall, and precision) of different machine learning algorithms using feature extraction approaches (VGG16, VGG19, ResNet50, and ResNet50V2) are displayed
  - When employing more sophisticated feature extraction models such as ResNet50 and ResNet50V2, there is a clear enhancement in performance metrics across all methods, as opposed to using VGG16 and VGG19.
  - The stacking classifier regularly attains the highest accuracy and F1 score among all feature extraction models, demonstrating its efficacy in amalgamating the capabilities of numerous base learners
  - Logistic regression, linear discriminant analysis (LDA), and support vector classifier (SVC) provide strong and consistent performance with various feature extraction models
(ii) Analysis of the differences between the whole feature and selected feature include the following:
  - Significant performance disparities are evident when comparing the outcomes of the initial four tables, which consist of VGG16 and VGG19, with the last four tables, which consist of ResNet50V2
  - Algorithms that employ ResNet50 and ResNet50V2 for feature extraction consistently achieve better results than those employing VGG16 and VGG19 across all performance criteria
  - The ResNet-based models excel due to their increased depth and exceptional capability to extract subtle information from medical pictures, particularly in challenging tasks such as prostate cancer diagnosis
  - Gaussian Naive Bayes (Gaussian NB) typically demonstrates inferior performance relative to other algorithms across all feature extraction models, indicating its limits in capturing the underlying patterns in the dataset
(iii) Implications and future directions include the following:
  - The findings emphasize the significance of selecting suitable feature extraction models for medical image analysis tasks, whereas more sophisticated architectures such as ResNet50 and ResNet50V2 provide greater performance
  - Subsequent investigations may delve into ensemble approaches and further optimization tactics to augment the efficacy of machine learning algorithms in detecting prostate cancer, capitalizing on the advantages of diverse feature extraction models
  - In addition, exploring transfer learning and fine-tuning techniques using pretrained models may offer valuable insights for enhancing the generalization and adaptability of models to various datasets
(iv) Highlights the superior performance reseasoning of ResNet-based models compared to VGG-based models include the following:
  - There exist notable architectural distinctions between ResNet (residual network) and VGG (visual geometry group). The ResNet architecture incorporates the notion of residual connections, enabling the model to acquire intrinsic functions rather than immediately acquiring the intended mappings. The utilization of ResNet allows for the efficient management of deeper network topologies, circumventing the issue of vanishing gradients commonly observed in conventional deep networks such as VGG.
  - ResNet is characterized by its deeper network designs, which have a greater number of layers in comparison to VGG. The capacity of ResNet to effectively train networks with significant depth enables it to effectively capture intricate features and patterns within the data, hence resulting in enhanced performance.
  - The residual connections of ResNet enhance the process of feature extraction by allowing the network to acquire residual representations. This feature aids in the preservation of crucial information across many levels of the network, enabling ResNet to effectively capture more intricate details and subtleties in the data as compared to VGG.

## 5. Limitation of the Research

Despite this research significantly advancing the use of supervised machine learning algorithms for prostate cancer detection, there are several important caveats to consider:

- Dataset diversity: The algorithm's capacity to apply to varied patient groups may be limited due to the lack of diversity in publically available datasets.
- Model interpretability is hindered by the intricate nature of supervised machine learning models, which presents difficulties in comprehending the acquired relationships. Consequently, this hampers the seamless incorporation of these models into clinical practice for physicians.
- Overfitting risk: The utilization of complicated algorithms amplifies the likelihood of overfitting to the training data, thus requiring comprehensive validation on varied datasets to assure the ability to generalize.
- Addressing ethical and regulatory concerns, including patient privacy and compliance with healthcare standards, is essential for the proper integration of machine learning in healthcare. This presents hurdles for its practical implementation.

## 6. Practical Implications

This research paper examines the practical consequences of using supervised machine learning algorithms to identify prostate cancer. The study seeks to enhance diagnostic precision and efficacy in the early detection of prostate cancer. Utilizing machine learning models, which have been trained on extensive datasets, yields favorable outcomes when compared to conventional diagnostic techniques [25]. The practical ramifications of integrating supervised machine learning algorithms for prostate cancer screening encompass:

- Utilizing supervised machine learning allows for the prompt detection of prostate cancer, leading to enhanced treatment results by identifying subtle patterns that may not be readily apparent using traditional methods.
- Machine learning methods enhance diagnostic precision and reliability by identifying subtle linkages among diagnostic parameters, resulting in a reduction of false positives and negatives.
- Personalized treatment strategies: Machine learning enables the development of customized treatment plans that consider the distinctive attributes of each patient, hence enhancing treatment efficacy and minimizing negative outcomes.
- Optimizing healthcare resources: By improving diagnostic precision, we may prioritize patients with a greater likelihood of having prostate cancer. This can result in cost savings and more effective distribution of healthcare services.

## 7. Future Works

The future of detecting prostate cancer relies on harnessing the advancing capabilities of machine learning algorithms. The following are essential domains for future investigation:

- Multimodal fusion: The integration of many types of data, such as biopsies, MRI imaging, and genomes, can provide a comprehensive understanding, potentially enhancing the accuracy of diagnosis.
- Customized risk evaluation: Adapting models to individual risk profiles, considering genetics, lifestyle, and medical history, could facilitate the implementation of focused screening and preventative strategies.
- Revealing concealed biomarkers: Machine learning could analyze extensive datasets and uncover new patterns of biomarkers that can be used for noninvasive and exceptionally accurate early detection.
- Utilizing artificial intelligence algorithms in clinical workflows might aid healthcare practitioners in analyzing data, suggesting treatment alternatives, and enhancing patient care routes.
- Mitigating algorithmic bias: It is essential to acknowledge and rectify any potential biases present in the training data and algorithms to provide fair and unbiased access to precise diagnosis for all patients.

## Figures and Tables

**Table 1:** MRI dataset statistics.

Corpus attribute	Attribute value
Total no. of healthy prostate	738
Total no. of infected prostate	3,514
Total no. of MRI prostate	4,252
Total no. of original images	22,036
Total no. of human subject	26
Original image size	(176, 176), (256, 256), and (512, 512)
Resize image	(224, 224)
Gray level depth of the images	24

**Table 2:** Statistical summary of logistic regression (all features).

Model	Accuracy (%)	F1 score (%)	Recall (%)	Precision (%)
VGG16	98.48	98.47	97.28	98.47
VGG19	99.64	99.64	99.11	99.64
ResNet50	99.52	99.52	98.82	99.53
ResNet50V2	99.41	99.41	98.76	99.41

**Table 3:** Statistical summary of decision tree classifier.

Model	Accuracy (%)	F1 score (%)	Recall (%)	Precision (%)
VGG16	96.23	96.16	92.13	96.21
VGG19	94.24	94.24	91.10	94.26
ResNet50	94.94	94.89	90.89	94.87
ResNet50V2	97.89	97.88	96.26	97.88

**Table 4:** Statistical summary of Gaussian NB.

Model	Accuracy (%)	F1 score (%)	Recall (%)	Precision (%)
VGG16	85.31	86.30	86.62	89.20
VGG19	84.13	85.28	85.68	88.60
ResNet50	93.07	93.20	91.25	93.47
ResNet50V2	84.96	86.10	87.96	89.88

**Table 5:** Statistical summary of K-neighbors classifier.

Model	Accuracy (%)	F1 score (%)	Recall (%)	Precision (%)
VGG16	98.70	98.70	97.20	98.70
VGG19	98.70	98.70	97.42	98.70
ResNet50	98.94	98.93	97.80	98.94
ResNet50V2	98.82	98.81	97.50	98.82

**Table 6:** Statistical summary of linear discriminant analysis.

Model	Accuracy (%)	F1 score (%)	Recall (%)	Precision (%)
VGG16	95.89	95.96	95.44	96.13
VGG19	96.00	96.05	95.07	96.16
ResNet50	99.41	99.41	98.98	99.41
ResNet50V2	97.53	97.56	96.91	97.59

**Table 7:** Statistical summary of SVC.

Model	Accuracy (%)	F1 score (%)	Recall (%)	Precision (%)
VGG16	97.30	97.23	93.68	97.33
VGG19	97.77	97.71	94.41	97.82
ResNet50	98.70	98.70	96.77	98.72
ResNet50V2	98.23	98.20	95.59	98.28

**Table 8:** Statistical summary of stacking classifier.

Model	Accuracy (%)	F1 score (%)	Recall (%)	Precision (%)
VGG16	98.48	98.47	97.28	98.47
VGG19	99.05	99.05	97.64	99.07
ResNet50	99.77	99.77	99.41	99.77
ResNet50V2	99.52	99.52	98.82	99.53

**Table 9:** Statistical summary of logistic regression.

Model	Accuracy (%)	F1 score (%)	Recall (%)	Precision (%)
VGG16	99.05	99.05	98.09	99.05
VGG19	99.41	99.41	98.76	99.41
ResNet50	99.41	99.41	98.76	99.41
ResNet50V2	99.52	99.52	98.82	99.53

**Table 10:** Statistical feature analysis.

Algorithm	Before feature extraction	Reduced features
Logistic regression	4,253	714
DT classifier	4,253	698
Gaussian NB	4,253	708
K-neighbors classifier	4,253	668
LDA	4,253	692
SVC	4,253	695
Stacking classifier	4,253	684

**Table 11:** Statistical summary of the VGG16 model (all features).

Algorithm	Accuracy (%)	F1 score (%)	Recall (%)	Precision (%)
Logistic regression	98.47	98.47	97.28	98.47
DT classifier	96.23	96.15	92.13	96.21
Gaussian NB	85.31	86.30	86.62	89.20
K-neighbors classifier	98.70	98.70	97.20	98.70
LDA	95.89	95.96	95.44	96.13
SVC	97.30	97.23	93.68	97.33
Stacking classifier	98.48	98.47	97.27	98.47

**Table 12:** Statistical summary of the VGG19 model (all features).

Algorithm	Accuracy (%)	F1 score (%)	Recall (%)	Precision (%)
Logistic regression	99.64	99.64	99.11	99.64
DT classifier	94.24	94.24	91.10	94.25
Gaussian NB	84.13	85.28	85.68	88.60
K-neighbors classifier	98.70	98.70	97.42	98.70
LDA	96.00	96.05	95.08	96.15
SVC	97.77	97.71	94.41	97.82
Stacking classifier	99.05	99.05	97.64	99.06

**Table 13:** Statistical summary of the ResNet50 model (all features).

Algorithm	Accuracy (%)	F1 score (%)	Recall (%)	Precision (%)
Logistic regression	99.52	99.52	98.82	99.53
DT classifier	94.94	94.89	90.89	94.87
Gaussian NB	93.07	93.20	91.25	93.47
K-neighbors classifier	98.94	98.93	97.80	98.94
LDA	99.41	99.41	98.98	99.41
SVC	98.70	98.70	96.77	98.72
Stacking classifier	99.77	99.77	99.41	99.77

**Table 14:** Statistical summary of the ResNet50V2 model (all features).

Algorithm	Accuracy (%)	F1 score (%)	Recall (%)	Precision (%)
Logistic regression	99.41	99.41	98.75	99.41
DT classifier	97.88	97.88	96.25	97.88
Gaussian NB	84.95	86.09	87.95	89.87
K-neighbors classifier	98.82	98.81	97.50	98.82
LDA	97.53	97.56	96.91	97.59
SVC	98.23	98.20	95.59	98.28
Stacking classifier	99.52	99.52	98.82	99.53

**Table 15:** Statistical summary of the VGG16 model (reduced features).

Algorithm	Accuracy (%)	F1 score (%)	Recall (%)	Precision (%)
Logistic regression	98.59	98.59	97.13	98.59
DT classifier	95.88	95.83	92.35	95.83
Gaussian NB	94.94	94.90	91.10	94.88
K-neighbors classifier	98.70	98.70	96.99	98.71
LDA	98.00	98.00	96.54	98.00
SVC	98.70	98.70	96.77	98.72
Stacking classifier	99.05	99.05	98.08	99.05

**Table 16:** Statistical summary of the VGG19 model (reduced features).

Algorithm	Accuracy (%)	F1 score (%)	Recall (%)	Precision (%)
Logistic regression	99.41	99.41	98.76	99.41
DT classifier	94.82	94.79	91.03	94.77
Gaussian NB	94.82	94.80	91.26	94.78
K-neighbors classifier	98.94	98.93	97.80	98.94
LDA	98.59	98.59	98.01	98.60
SVC	99.52	99.52	98.82	99.53
Stacking classifier	99.41	99.41	98.76	99.41

**Table 17:** Statistical summary of the ResNet50 model (reduced features).

Algorithm	Accuracy (%)	F1 score (%)	Recall (%)	Precision (%)
Logistic regression	99.41	99.41	98.76	99.41
DT classifier	94.82	94.76	90.59	94.74
Gaussian NB	97.17	97.11	93.38	97.22
K-neighbors classifier	99.30	99.30	98.46	99.30
LDA	99.30	99.30	98.68	99.30
SVC	99.30	99.30	98.23	99.30
Stacking classifier	99.64	99.64	99.11	99.64

**Table 18:** Statistical summary of the ResNet50V2 model (reduced features).

Algorithm	Accuracy (%)	F1 score (%)	Recall (%)	Precision (%)
Logistic regression	99.52	99.52	98.82	99.53
DT classifier	98.11	98.11	96.61	98.10
Gaussian NB	93.53	93.43	88.46	93.40
K-neighbors classifier	99.30	99.30	98.68	99.30
LDA	99.41	99.41	98.98	99.41
SVC	99.52	99.52	98.82	99.53
Stacking classifier	99.52	99.52	99.26	99.52

## Data Availability

The prostate-MRI data used to support the findings of this study could be found in the repository DOI: 10.7937/K9/TCIA.2016.6046GUDv.
